# Red Araça (*Psidium cattleianum*) Extract Prevents Right Ventricular Hypertrophy in Experimental Pulmonary Arterial Hypertension

**DOI:** 10.3390/biomedicines14091939

**Published:** 2026-08-29

**Authors:** Custódio José Gaspar, Patrick Turck, Alan Bahr, Alexsandra Zimmer, Daniela Drosdowski, Tânia Regina G. Fernandes, Elissa Fernandes, Sílvio Tasca, Vitória Rosa de Oliveira, Felipe Hiran Lino da Silva, Cristina Campos Carraro, Rodrigo Binkowski de Andrade, Eliseu Rodrigues, Carlos Henrique Pagno, Rita de Kássia Souza, Fernanda Visioli, Adriane Belló-Klein, Katya Vianna Rigatto, Alex Sander da Rosa Araujo, Alexandre Luz de Castro

**Affiliations:** 1 Laboratory of Cardiovascular Physiology and Reactive Oxygen Species, Physiology Department, Institute of Basic Health Science (ICBS), Federal University of Rio Grande do Sul (UFRGS), Ramiro Barcelos Street, 2600-Santa Cecília, Porto Alegre 90035-003, Rio Grande do Sul, Brazil; 2 Food Science and Technology Institute, Federal University of Rio Grande do Sul (UFRGS), Avenue Bento Gonçalves n° 9500, Porto Alegre 91501-970, Rio Grande do Sul, Brazil; 3 Faculty of Dentistry, Federal University of Rio Grande do Sul (UFRGS), Ramiro Barcelos Street, 2492-Santa Cecília, Porto Alegre 90035-004, Rio Grande do Sul, Brazil; 4 Department of Basic Health Sciences, Federal University of Health Sciences of Porto Alegre (UFCSPA) Sarmento Leite Street, 245, Porto Alegre 90050-170, Rio Grande do Sul, Brazil

**Keywords:** red araça, lung fibrosis, pulmonary hypertension, right ventricle

## Abstract

**Background:** Pulmonary artery hypertension (PAH) is characterised by progressive pulmonary vascular remodelling and increased pulmonary vascular resistance, leading to right ventricular (RV) dysfunction and failure. Red araça (*Psidium cattleianum*) extract is derived from native Brazilian fruit rich in phenolic compounds with recognised antioxidant and anti-inflammatory properties. **Objectives:** The present study aimed to evaluate the preventive effects of red araça extract administration on RV remodelling and pulmonary arteriole alterations in rats with MCT-induced PAH. **Methods:** Male Wistar rats were divided into three groups: control, monocrotaline (MCT), and MCT + red araça extract. PAH was induced by a single intraperitoneal injection of MCT (60 mg/kg), and the treated group received red araça extract (5 mL/kg/day) by gavage for 21 days. Echocardiographic, hemodynamic, morphometric, molecular, and histological analyses were subsequently performed. **Results:** The MCT group exhibited increased pulmonary vascular resistance and RV hypertrophy, impaired RV function, elevated TLR4 and xanthine oxidative (XO) protein expression, elevated Akt activation, and marked increase in pulmonary arteriole wall thickness. Treatment with red araça extract prevented RV hypertrophy, reduced total pulmonary vascular resistance, preserved Akt activation similar to control, and decreased TLR4 and XO protein expression in the RV. In addition, administration of red araça extract prevented the increase in pulmonary arteriolar wall thickness. **Conclusions:** Collectively, these findings suggest that red araça extract attenuates cardiopulmonary alterations associated with experimental PAH, possibly involving modulation of the expression of proteins related to cardiac hypertrophy and inflammation.

## 1. Introduction

Pulmonary artery hypertension (PAH) is a heterogeneous and progressive cardiopulmonary disease characterised by pulmonary vascular remodelling and increased pulmonary vascular resistance, ultimately leading to right ventricular (RV) dysfunction and failure [1,2,3,4]. Hemodynamically, PAH is defined by a mean pulmonary artery pressure (mPAP) ≥ 20 mmHg associated with pulmonary vascular resistance ≥2 Wood units [1,2]. Sustained RV pressure overload initially induces compensatory hypertrophy; however, persistent overload progressively contributes to maladaptive remodelling, ventricular dysfunction, and heart failure, which represent major determinants of prognosis in PAH [1,2,3,4].

The important cardiopulmonary alterations observed in PAH can be reproduced with an experimental model in which animals are injected with monocrotaline (MCT) [5]. This model promotes pulmonary vascular injury associated with inflammation, fibrosis, endothelial dysfunction, and increased pulmonary vascular resistance, resulting in progressive RV overload and dysfunction [5,6]. In addition to pulmonary vascular alterations, inflammatory signalling pathways have emerged as important contributors to RV remodelling during PAH progression [7]. Among these pathways, Toll-like receptor 4 (TLR4) has received increasing attention due to its role in activation of innate immune responses and production of pro-inflammatory mediators associated with tissue injury and fibrosis [7,8,9,10]. Increased TLR4 signalling has been associated with pathological cardiac remodelling and RV dysfunction in experimental PAH [7,10].

In parallel with inflammatory activation, increased expression of proteins related to cardiac hypertrophy also contribute to RV dysfunction in PAH. In this context, protein kinase B (Akt) signalling plays an important role in cardiomyocyte remodelling and cellular stress responses [11,12]. Previous studies demonstrated that increased Akt signalling is related to cardiac hypertrophy [13]. In addition, alterations in xanthine oxidase (XO) expression have also been described in cardiac disease [14]. Under physiological conditions, xanthine dehydrogenase form is predominant; however, it can be converted into XO by the oxidation of sulfhydryl residues or by limited proteolysis. This enzyme can generate hydrogen peroxide through the catalytic oxidative hydroxylation of purine substrates and promote oxidative injury and inflammation in the heart [14].

Despite advances in pharmacological therapies, there are limited treatment strategies for PAH. They primarily focus on slowing disease progression rather than reversing cardiopulmonary remodelling [2]. Therefore, the identification of novel therapeutic approaches capable of modulating inflammatory and remodelling-related pathways remains an important challenge. In this context, natural products rich in phenolic compounds have increasingly attracted attention due to their antioxidant and anti-inflammatory properties. Previous studies have demonstrated beneficial effects of compounds such as pterostilbene, sulforaphane, and blueberry extract in experimental PAH models [15,16].

Red araça (*Psidium cattleianum*), also known as strawberry guava, is a native Brazilian fruit rich in phenolic compounds, flavonoids, anthocyanins, carotenoids, and vitamin C [17,18]. Several of these compounds have been associated with antioxidant, anti-inflammatory, and cardioprotective activities [18]. In addition, previous studies have demonstrated anti-inflammatory and antiproliferative effects of red araça extracts, including modulation of matrix metalloproteinases and inflammatory signalling pathways [19,20]. However, to the best of our knowledge, no previous study has investigated the effects of red araça extract in experimental PAH. Therefore, the present study aimed to evaluate the preventive effects of red araça extract administration on RV remodelling and pulmonary arteriole alterations in rats with MCT-induced PAH. Functional, hemodynamic, morphometric, molecular, and histological parameters were evaluated to investigate the potential cardiopulmonary effects of this native Brazilian fruit in experimental PAH. We hypothesise that red araça extract has positive effects on the RV of rats with PAH.

## 2. Materials and Methods

### 2.1. Drugs and Reagents

Ketamine hydrochloride was purchased from König Lab S.A. (São Paulo, Brazil), and xylazine was purchased from Virbac do Brasil I.P. (São Paulo, Brazil). MCT and all other drugs and reagents were purchased from Sigma-Aldrich (St. Louis, MO, USA).

### 2.2. Preparation of Red Araça Extract

Red araça was donated by the Cooperative of Family Farming Producers of Passo Fundo city (COOPAF, Passo Fundo, Brazil). The samples were collected and transported to the laboratory at 5.0 ± 1.0 °C, where they were freeze-dried (Liotop^®^ L101, Vitória, Brazil), milled (HC-32P, Black & Decker, Inc., Newark, DE, USA) into a fine powder, vacuum-packed, and stored in a freezer prior to extraction. For the preparation of the red araça extract, 100 g of freeze-dried fruit was weighed and homogenised with 750 mL of distilled water. The mixture was centrifuged (1000× *g* for 15 min at 4 °C; HITACHI High-Speed Refrigerated CR21GIII, Ibaraki, Japan), and approximately 550 mL of the supernatant was collected and stored at −18 °C [21].

### 2.3. Evaluation of the Composition of Red Araça Extract

To evaluate the composition of red araça extract, 400 µL of the obtained extract was homogenised with 600 µL of high-performance liquid chromatography (HPLC)-grade methanol in an Eppendorf tube, vortexed for 3 min (USC-1400, Unique), and then centrifuged at 3000× *g* for 5 min. The supernatant was filtered through a 0.22-µm cellulose acetate membrane and subjected to analysis by ultra-high-performance liquid chromatography–electrospray ionisation–quadrupole time-of-flight tandem mass spectrometry (HPLC-ESI-QTOF-MS/MS) using a Nexera X2 UHPLC system (Shimadzu, Kyoto, Japan), connected to a mass spectrometer equipped with QTOF analyser. An electrospray ionisation (ESI) source (Impact II, Bruker, MA, USA) was employed as the ionisation source. Samples were analysed in the negative and positive electrospray modes.

The separation was performed using a C18 Acquity UPLC T3 column (50 × 2.1 mm, 1.8 μm particle size) at a flow rate of 0.5 mL min^−1^ and a temperature of 40 °C. The mobile phase consisted of solvent A (water acidified with 0.1% formic acid) and solvent B (acetonitrile acidified with 0.1% formic acid). The mobile-phase gradient was as follows: 0% B for 0.5 min, followed by a linear increase to 100% B over 8 min, 100% B for 1 min, and a return to 0% B for an additional 1 min. The injection volume was 5 μL.

The QTOF-MS parameters were as follows: a scan range of *m*/*z* 50–1500, a capillary voltage of 3500 V in the positive-ion mode and −3500 V in the negative-ion mode, a drying-gas temperature of 310 °C, and a nebuliser gas pressure of 4 bar. MS/MS spectra were acquired in data-dependent acquisition mode with a threshold of 1500. Data Analysis 4.3 (Bruker Daltonics, Billerica, MA, USA) was used for instrument control and raw-data processing. Raw data were internally calibrated using sodium formate clusters. Compound annotation was performed by comparing the fragmentation data obtained from the samples with spectral libraries, fragmentation data reported in the literature, the GNPS2 online platform (https://gnps2.org/) and SIRIUS (https://bio.informatik.uni-jena.de/software/sirius/, accessed on 10 April 2024) [21]. Compound identification confidence levels were assigned in accordance with the criteria established by Schymanski et al. [22], ranging from confirmed structure (Level 1, using authentic standards) and probable structure (Level 2, based on library and literature spectral matching) to tentative candidate (Level 3, based on structure predicted by in silico tools such as SIRIUS).

### 2.4. Quantification of the Total Phenol Content

The total phenolic content was determined based on absorbance at 765 nm using a spectrophotometer (8452A, Hewlett-Packard, Florianópolis, Brazil) using the Folin–Ciocalteu method [23]. It is presented as milligrams of gallic acid equivalents per litre (mg GAE/L).

### 2.5. Animals and Experimental Groups

All procedures were approved by the institutional animal ethics committee (project approval number 45303). In total, 30 male Wistar rats (weighing 170–260 g) were obtained from the Central Animal House of the Federal University of Rio Grande do Sul (UFRGS). They were maintained under standard conditions (a controlled temperature of 21 °C and a 12-h photoperiod). All animals had *ad libitum* access to water and regular rodent chow (see Table 1 for the nutritional profile), and the experiments were conducted in accordance with institutional guidelines and the Guide for the Care and Use of Laboratory Animals (US Department of Health and Human Services, NIH Publication No. 86-23).

The rats were randomly assigned to one of three experimental groups as soon as they arrived at the experimental animal facility: control (*n* = 10), MCT (*n* = 10), or MCT + red araça extract (*n* = 10). PAH was induced in the MCT and MCT + red araça extract groups by a single intraperitoneal injection of MCT (60 mg/kg body weight). The control group received an intraperitoneal injection of saline [24]. After PAH induction, the experimental period lasted 21 days. The control and MCT groups received saline via gavage once a day, while the MCT + red araça extract group received red araça extract (5 mL/kg body weight) via gavage once a day. The volume given by gavage was the same for the animals in all three groups. This dose of red araçá extract was based on a previous study that evaluated the estimated amount of polyphenols consumed daily by the Brazilian population [25]. The extract had a total phenolic content of 376.16 mg GAE/L (determined as described in Section 2.4), so a dose of 5 mL/kg/day is equivalent to a daily intake of 6.8 mg of phenolic compounds. Moreover, this administration of red araçá extract was preventive given that it was administered while PAH developed.

The rats were assessed daily. Rats showing changes in posture (an inability to stand, arching of the back), mucous membrane colour (cyanosis, paleness), pain obviously present rated as ‘2’ according to the Grimace scale, dyspnoea (through observation of increased respiratory rate), reduced movement (less exploration of the cage, staying lying down or in the same spot for a long time compared to other animals in the same experimental group), significant weight reduction compared with other rats in the same experimental group, and signs of infection would be euthanised. However, none of the rats showed these signs, so no rats were excluded or euthanised during the 21-day experiment.

### 2.6. Echocardiographic Analysis

Twenty-one days after MCT injection, echocardiography was performed. The rats were anaesthetised with an intraperitoneal injection of 90 mg/kg body weight ketamine and 20 mg/kg body weight xylazine [16] and placed in the left lateral decubitus position to obtain cardiac images. All echocardiographic evaluations were performed using the EnVisor system (Philips, Andover, MA, USA) and a 2–13 MHz transducer by the same trained operator—who had experience in small animal echocardiography and was blinded to the treatment of each rat. All transthoracic echocardiographic parameters and calculations were performed according to the recommendations published by the American Society of Echocardiography. All images were evaluated during the examination and also assessed offline, using Sante DICOM viewer (Santesoft LTD, Athens, Greece), by another echocardiographer, also blinded to the treatment of each rat, to assess interobserver variability.

The following parameters were obtained from two-dimensional images and M-mode tracings: RV systolic output, heart rate, and RV systolic and diastolic areas. Fractional area change (FAC) was calculated as follows: (RV diastolic area − RV systolic area)/RV diastolic area × 100 and expressed as a percentage. Tricuspid annular plane systolic excursion (TAPSE) was also measured as an indicator of RV systolic function and contractility. TAPSE represents the displacement of the tricuspid annulus toward the RV apex during systole [26,27,28].

Flow–velocity curves obtained by pulsed-wave Doppler were also evaluated. The peak E and peak A velocities across the tricuspid valve were measured as indicators of RV diastolic function. Peak E represents the rapid filling phase of the cardiac cycle, whereas the interval between the E and A waves corresponds to the slow filling phase (diastasis). Peak A represents ventricular filling resulting from atrial systole. Heart rate was calculated from the time interval between two consecutive RV ejection-flow waves measured by Doppler [26,27,28].

### 2.7. Evaluation of Hemodynamic Parameters

RV and pulmonary artery haemodynamic parameters were assessed 21 days after MCT injection. The rats were anaesthetised with an intraperitoneal injection of 90 mg/kg body weight ketamine and 20 mg/kg body weight xylazine. A PE-50 catheter connected to a transducer (Miniature Pulse Transducer PR-155, Narco Biosystems, Houston, TX, USA) and a pressure amplifier (HP 8805C, Hewlett-Packard, Andover, MA, USA) was inserted through the right jugular vein into the RV. Pressure data, recorded in millimetres of mercury (mmHg), were digitised using the WinDaq Data Acquisition System (Dataq Instruments Inc., Akron, OH, USA) at a sampling frequency of 1000 Hz.

mPAP was estimated using the following equation: mPAP (mmHg) = 0.61 × RVSP + 2 mmHg, where RVSP denotes RV systolic pressure. Total pulmonary vascular resistance (TPVR) was calculated according to Koskenvuo et al. [29] using the following equation: TPVR = 80 × mPAP (mmHg)/cardiac output (L). This indirect estimate of TPVR has been shown to correlate strongly with direct invasive measurements of this parameter [29].

### 2.8. Morphometric Analysis

After hemodynamic evaluation, rats were killed by decapitation and the RV was harvested for morphometric, biochemical, and Western blot analyses. The RV and left ventricle (LV) were weighed to determine ventricular hypertrophy indexes: the RV weight-to-body ratio, calculated as RV weight/body weight, and the Fulton index, calculated as RV weight/(LV weight + septum weight). The lungs and liver were also collected and weighed. Liver and right lung weights were used to assess congestion based on the organ-weight-to-body-weight ratio [27,30]. The left lung was used for histological evaluation.

### 2.9. Preparation of RV Homogenates

The RV was rapidly frozen in liquid nitrogen and stored at −80 °C until Western blot analysis. RV tissue was homogenised for 30 s with an Ultra-Turrax homogeniser (OMNI Tissue Homogenizer, OMNI International, USA) in the presence of 1.15% KCl (5 mL/g tissue) and 100 mmol/L phenylmethylsulphonyl fluoride. The samples were centrifuged at 10,000× *g* for 20 min at 4 °C, and the supernatant was collected and stored at −80 °C for further analyses [31]. The protein concentration was determined using the Lowry method, with bovine serum albumin as the standard [32].

### 2.10. Serum Parameters of Kidney Function and Liver Damage

After collection, blood was centrifuged at 1000× *g* for 10 min at 4 °C, and serum was removed to measure liver enzymes (aspartate aminotransferase [AST] and alanine aminotransferase [ALT]), urea and creatinine using a kit for the Alinity C analyser (Abbott, São Paulo, Brazil).

### 2.11. Western Blotting

Following RV homogenisation, 100 μg of protein from each sample was subjected to one-dimensional polyacrylamide gel electrophoresis under denaturing conditions using sodium dodecyl sulfate in a discontinuous system, with 8–14% (*w/v*) resolving and stacking gels [33]. The following primary antibodies were used for protein detection (all from Santa Cruz Biotechnology, Santa Cruz, CA, USA): TLR4 (90 kDa; sc-293072; 1:1000), Akt (62 kDa; sc-5298; 1:1000), p-Akt (62kDa; sc-514032) and XO (150 kDa; sc-398548; 1:1000). Primary antibody binding was detected using a horseradish peroxidase-conjugated anti-rabbit secondary antibody. Chemiluminescent reagents were used to develop the membranes, and the resulting images were quantitatively analysed using ImageJ version 1.47t (National Institutes of Health, Bethesda, MD, USA). A molecular-weight marker (RPN 800 Rainbow Full Range, Bio-Rad, Hercules, CA, USA) was used as a reference for determining the molecular weights of the bands. Protein loading was normalised using Ponceau S staining.

### 2.12. Histological Analysis

The left lungs were fixed in 10% paraformaldehyde, dehydrated through graded alcohol and xylene, and embedded in paraffin. After cooling, the paraffin blocks were sectioned at a thickness of 3 μm using a microtome. The sections were mounted on slides, deparaffinised, and stained with haematoxylin and eosin (H&E). The slides were examined using a binocular microscope equipped with a QColor 5 camera at 200× magnification (Olympus, Hachioji, Japan).

The external diameter of each arteriole was defined as the external vascular diameter, whereas the diameter of the vessel lumen was defined as the internal vascular diameter. Only pulmonary arterioles with an internal diameter < 200 μm were evaluated. Vascular wall thickness was estimated by subtracting the internal diameter from the external diameter. Measurements were performed using Fiji/ImageJ (National Institutes of Health). The histological analysis was performed by a pathologist who was blinded to the experimental groups [33].

### 2.13. Sample Size Calculation and Statistical Analysis

For the sample size calculation, an α level of 0.05 and a statistical power of 0.95 (1 − β) were assumed. The effect size was estimated as 0.85. The sample size was calculated for the HAP protocol based on an expected improvement in TAPSE, a measure of RV function [24]. Using G*Power software version 3.1, a total sample size of 30 animals was calculated (n = 10 per group). Except for the Western blot analysis, which included four animals per group, all other analyses were performed using samples from all animals (n = 10 per group).

Data normality was assessed using the Shapiro–Wilk test. Parametric data are presented as mean ± standard deviation and were analysed using one-way analysis of variance (ANOVA), followed by the Student–Newman–Keuls post hoc test. Nonparametric data are presented as median (interquartile range) and were analysed using the Kruskal–Wallis test, followed by Dunn’s post hoc test. Differences were considered statistically significant at *p* < 0.05. Statistical analyses were performed using SigmaPlot version 12.0, and graphs were generated using GraphPad Prism version 5.0 (GraphPad Software, San Diego, CA, USA).

## 3. Results

### 3.1. Composition of Red Araça Extract

The total phenolic content of the red araça extract was 1376.16 mg GAE/L. The main compounds identified by HPLC-ESI-QTOF-MS/MS are presented in Table 2.

### 3.2. Echocardiographic Assessment of RV Function

**Figure 1:**
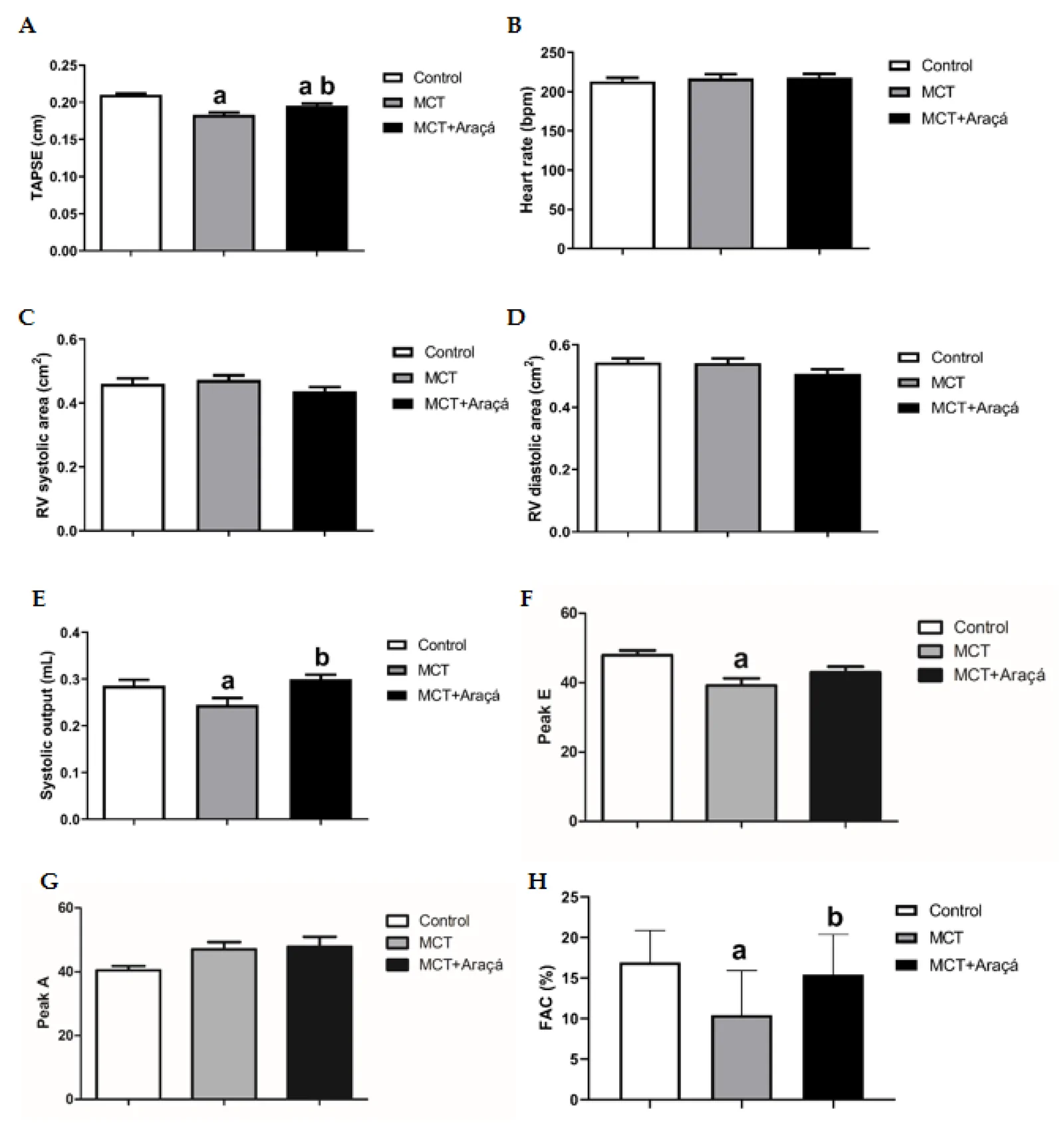
Red araça extract improves right ventricular (RV) functional parameters in rats with pulmonary artery hypotension (PAH). Echocardiographic analyses were performed in the control, monocrotaline (MCT), and MCT + red araça extract groups. Representative echocardiographic parameters of (**A**) tricuspid annular plane systolic excursion (TAPSE) (*p* < 0.0001), (**B**) heart rate (beats per minute), (**C**) RV systolic area, (**D**) RV diastolic area, (**E**) RV systolic output (*p* = 0.0121), (**F**) the peak E wave (*p* = 0.0047), (**G**) the peak A wave, and (**H**) the fractional area change (FAC, (%) (*p* = 0.0218). The data are presented as the mean ± standard deviation (*n* = 10 rats per group). ^a^ *p* < 0.05 versus the control group; ^b^ *p* < 0.05 versus the MCT group.

As shown in Figure 1, TAPSE was reduced in the MCT and MCT + red araça extract groups compared with the control group (*p* < 0.05). Moreover, TAPSE was higher in the MCT + red araça extract group compared with the MCT group (*p* < 0.05). RV systolic area, RV diastolic area, and heart rate did not differ significantly among the experimental groups. RV systolic output was reduced in the MCT group compared with the control group, whereas this reduction was prevented in the MCT + red araça extract group. The peak E wave was reduced in the MCT group compared with the control group (*p* < 0.05), but there was no difference in the parameter between the MCT + red araça extract and control groups. The peak A wave did not differ among the groups. The fractional area change (FAC) was reduced in the MCT group, and administration of red araça extract prevented this decrease.

### 3.3. RV Hemodynamic Parameters

RVSP, the RV relaxation index (−dP/dt), TPVR, and mPAP were increased in the MCT and MCT + red araça extract groups compared with the control group (Table 3). Moreover, TPVR was lower in the MCT + red araça extract group compared with the MCT group, indicating that partial attenuation of pulmonary vascular resistance increased with administration of red araça extract. RV end-diastolic pressure (RVEDP) and the RV contractility index (+dP/dt) did not differ among the experimental groups.

### 3.4. Morphometric Evaluation

**Figure 2:**
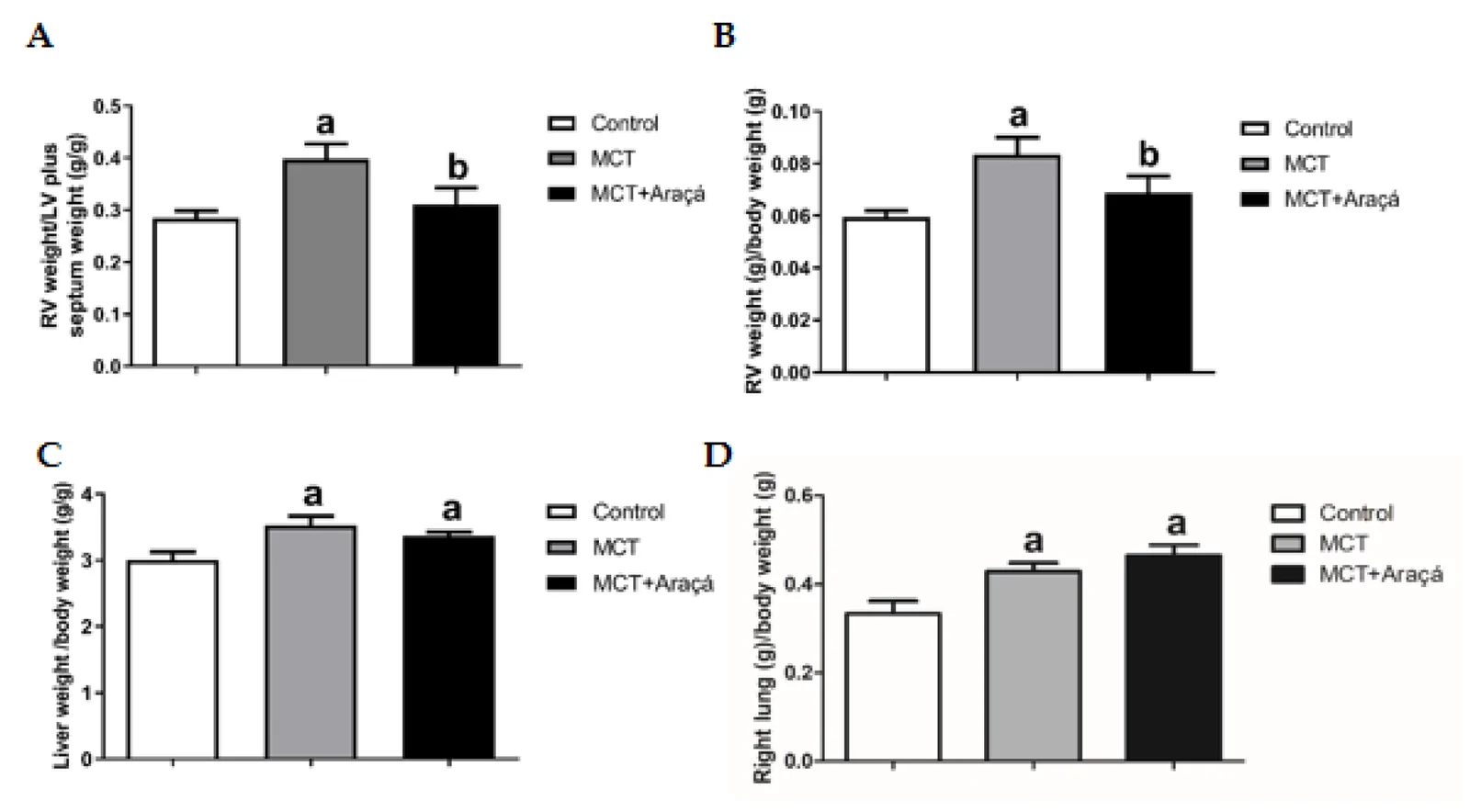
Red araça extract helps prevent right ventricular (RV) hypertrophy in rats with pulmonary artery hypertension (PAH). Morphometric analyses were performed in the control, monocrotaline (MCT), and MCT + red araça extract groups. Representative morphometric parameters of (**A**) the Fulton index, calculated as RV weight/(left ventricular weight + septum weight) (*p* = 0.0086), (**B**) the RV weight-to-body weight ratio (*p* = 0.0076), (**C**) the liver weight-to-body weight ratio (*p* = 0.0064), and (**D**) the lung weight-to-body weight ratio (*p* = 0.0002). The data are presented as the mean ± standard deviation (*n* = 10 rats per group). ^a^ *p* < 0.05 versus the control group; ^b^ *p* < 0.05 versus the MCT group.

The Fulton index was increased in the MCT group compared with the control group, but not in the MCT + red araça extract group. The RV weight-to-body weight ratio was increased in the MCT group compared with the control group (*p* < 0.05; Figure 2A). In contrast, it was not increased in the MCT + red araça extract group. The liver and lung weight-to-body weight ratios were increased in the MCT and MCT + red araça extract groups compared with the control group (*p* < 0.05; Figure 2B,C).

### 3.5. Biochemical Parameters of Kidney Function and Liver Damage

As shown in Table 4, the serum creatinine level was significantly higher in the MCT and MCT + red araça extract groups compared with the control group (*p* = 0.013). However, the serum urea, ALT, and AST levels did not differ among the groups.

### 3.6. TLR4, p-Akt/Akt, and XO Protein Expression in the RV

**Figure 3:**
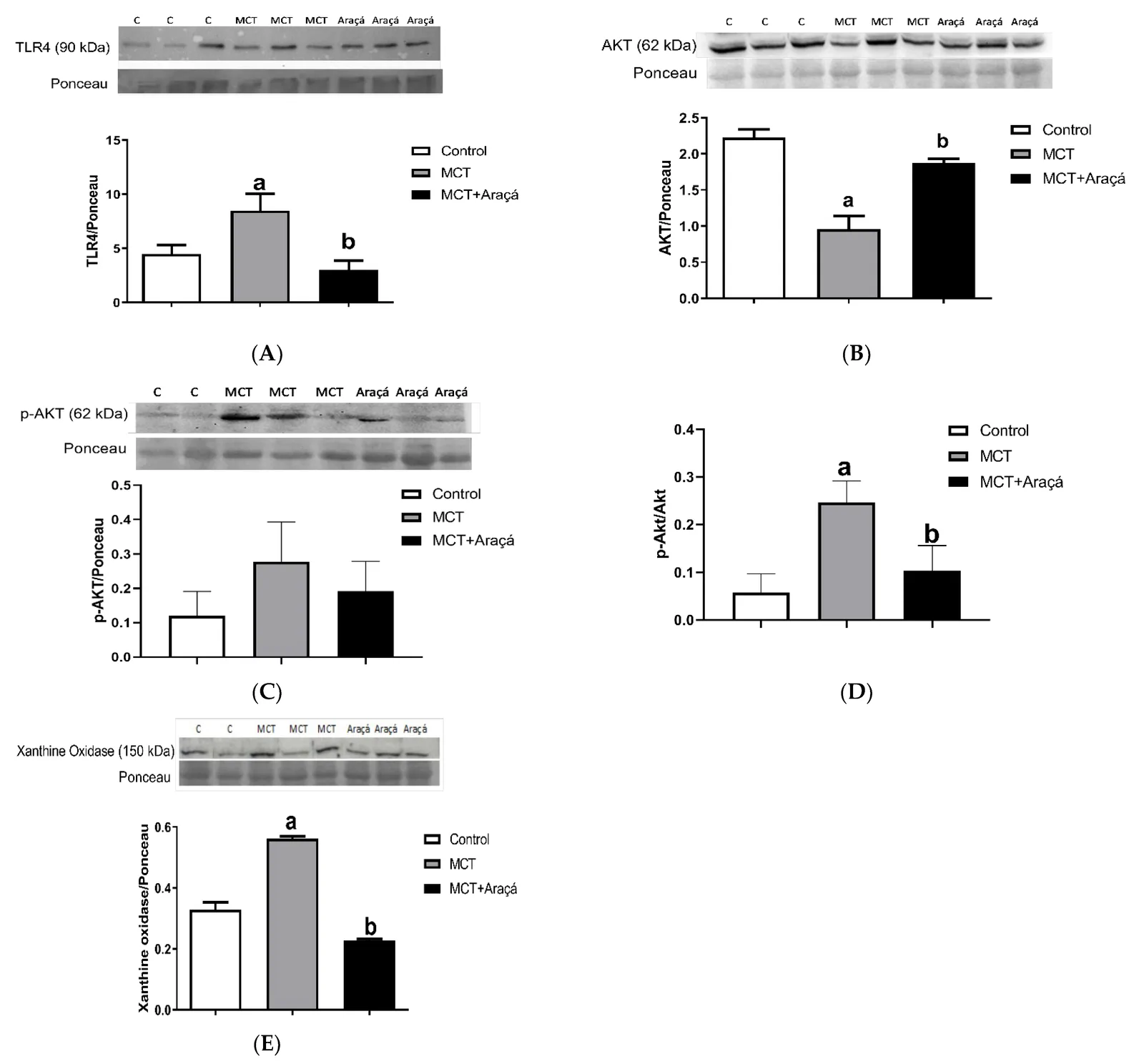
Red araça extract shifts molecular signalling towards a less inflammatory and more pro-survival profile in the right ventricular (RV) of rats with pulmonary artery hypertension (PAH). Western blotting was performed using RV lysates from the control, monocrotaline (MCT), and MCT + red araça extract groups. Representative Western blots (**top**) and quantitative analysis (**bottom**) of relative expression are shown for (**A**) Toll-like receptor 4 (TLR4) (*p* = 0.0103), (**B**) protein kinase B (Akt) (*p* = 0.0011), (**C**) p-Akt, (**D**) p-Akt/Akt (*p* = 0.0346), (**E**) xanthine oxidase (XO) (*p* = 0.0001). Protein expression was normalised to Ponceau staining. The data are presented as the mean ± standard deviation (*n* = 4 rats per group). ^a^ *p* < 0.05 versus the control group; ^b^ *p* < 0.05 versus the MCT group.

Western blotting demonstrated increased TLR4 protein expression in the RV of the MCT group compared with the control group. In contrast, TLR4 was decreased in the MCT + red araça extract group compared with the MCT group (*p* < 0.05), and there was no difference between the MCT + red araça extract and control groups (Figure 3A). Total Akt protein expression was markedly reduced in the MCT group compared with the control group. It was higher in the MCT + red araça extract group compared with the MCT group (*p* < 0.05) but did not differ from the control group (Figure 3B). p-Akt levels were not different among groups (Figure 3C). p-Akt/Akt ratio was increased in MCT group when compared with the control. In contrast, p-Akt/Akt ratio was decreased in the MCT + red araça extract group compared with the MCT group (*p* < 0.05), and there was no difference between the MCT + red araça extract and control groups (Figure 3D). Finally, XO protein expression was increased in MCT rats when compared with control and MCT + araça group (Figure 3E).

### 3.7. Evaluation of Pulmonary Arteriolar Wall Thickness

**Figure 4:**
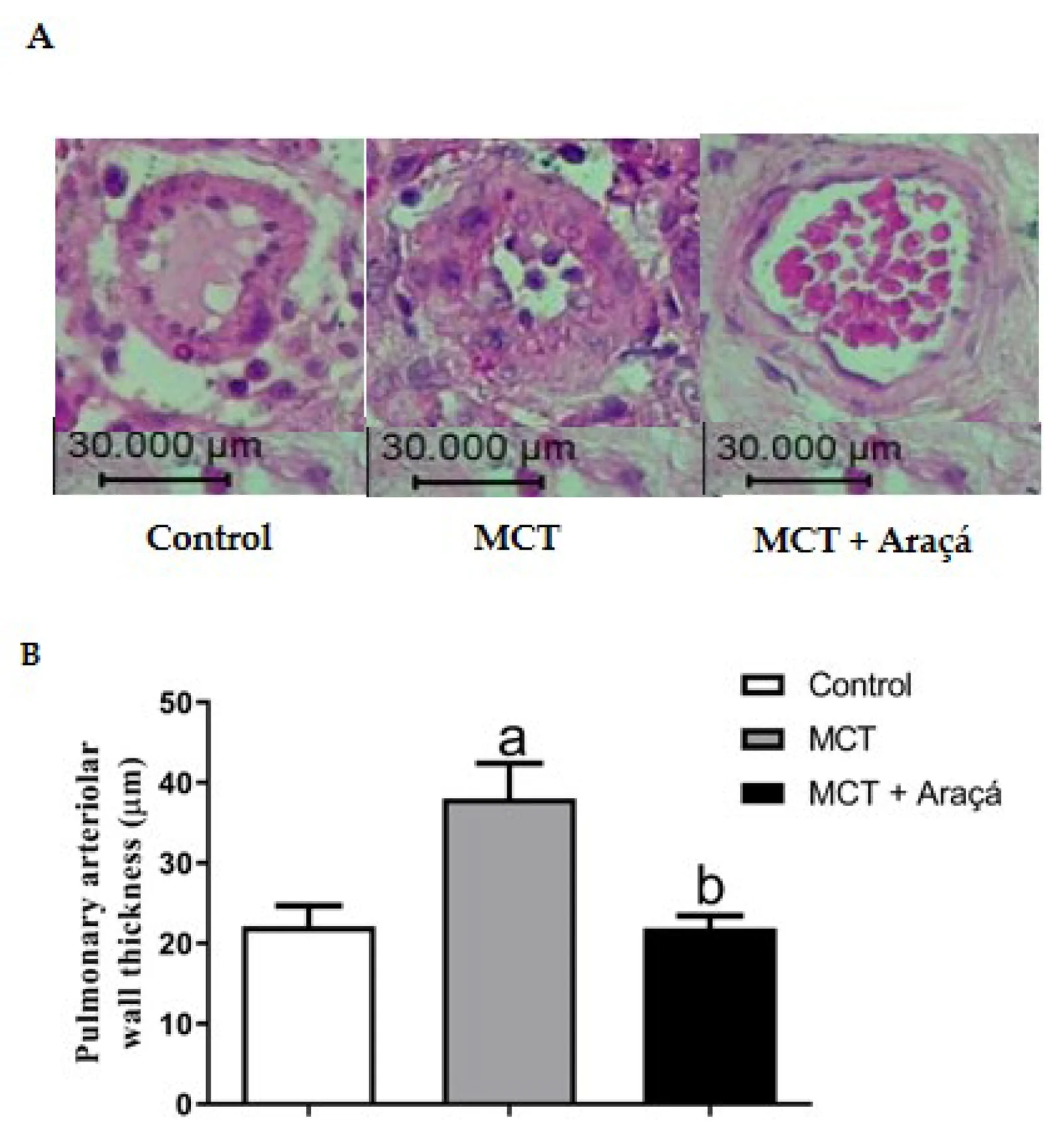
Red araça extract prevents the increase in pulmonary arteriolar wall thickness. (**A**) Representative photomicrographs of lung tissue sections showing pulmonary arterioles obtained from the control, monocrotaline (MCT), and MCT + red araça extract groups, and (**B**) pulmonary arteriolar wall thickness (µm) (*p* < 0.0001). The data are presented as the mean ± standard deviation (*n* = 10 rats per group). ^a^ *p* < 0.05 versus the control group; ^b^ *p* < 0.05 versus the MCT group.

Histological analyses demonstrated a marked increase in pulmonary arteriolar wall thickness in the MCT group. Administration of red araça extract prevented this increase (Figure 4).

## 4. Discussion

The present study investigated the effects of administration of red araça extract on RV remodelling and pulmonary arteriole alterations in rats with MCT-induced PAH. The results support the hypothesis that red araça extract has positive effects on the RV of rats with PAH. The major conclusions are that treatment with red araça extract attenuated RV dysfunction and prevented RV hypertrophy and the increase in pulmonary arteriole wall thickness. In addition, red araça extract modulated the expression of proteins associated with inflammation and cardiac hypertrophy, specifically a reduction in TLR4 and XO compared with the MCT group and preserved Akt activation in the RV. Together, these findings suggest that red araça extract may attenuate cardiopulmonary alterations associated with experimental PAH.

The red araça extract used in the present study exhibited a high total phenolic content and contained several bioactive compounds previously associated with antioxidant, anti-inflammatory, and cytoprotective activities. Among the compounds identified were catechin, epicatechin, quercetin derivatives, anthocyanins, daidzein, and pinocembrin, all of which have been associated with protective effects in cardiovascular and inflammatory conditions [18]. Previous studies have also demonstrated anti-inflammatory and antiproliferative properties of red araça extracts. In fact, a study observed that red araça leaf extract suppressed both matrix metalloproteinases (MMP)-9 and MMP-2 expression and activity in part through the downregulation of extracellular signal-regulated kinase (ERK)1/2 activation in lung cancer cells [19]. Medina et al. [20] showed that this extract had an anti-proliferative effect in human cancer cells. Although the present study was not designed to determine the individual contribution of each compound, the coexistence of multiple phenolic compounds with complementary biological properties may be relevant to the cardiopulmonary effects observed in the experimental model of MCT-induced PAH. Importantly, natural extracts rich in polyphenols frequently exert multitarget biological actions, simultaneously modulating inflammatory, oxidative, and remodelling-related pathways rather than acting through a single isolated mechanism. In relation to this, Turck and colleagues showed that blueberry extract has been shown to reduce hemodynamic changes and pulmonary arteriolar remodeling in rats with PAH, effects linked to preserved RV systolic function [34].

The MCT-induced PAH model reproduced key cardiopulmonary alterations typically observed in pulmonary hypertension. Rats treated with MCT exhibited increased RVSP, mPAP, and TPVR, indicating the establishment of pulmonary vascular dysfunction and sustained RV pressure overload [24]. These findings are consistent with previous studies demonstrating that administration of MCT promotes progressive pulmonary vascular injury and endothelial dysfunction and impairs pulmonary haemodynamics, ultimately resulting in increased RV afterload [5,24,27]. In the present study, the haemodynamic alterations observed in the MCT group were accompanied by structural and functional changes in the heart. RV hypertrophy associated with reduced TAPSE, FAC, and systolic output suggests the development of maladaptive ventricular remodelling and progressive impairment of contractile performance. Persistent pressure overload is known to initially induce compensatory hypertrophy aimed at preserving cardiac output; however, sustained overload progressively contributes to inflammatory activation, metabolic stress, fibrosis, and ventricular dysfunction during PAH progression [1,2,3,4]. In the present study, the reduction in RV functional parameters observed in the MCT group corroborates previous reports demonstrating deterioration of RV systolic performance in experimental PAH models [27].

In addition to cardiac alterations, pulmonary arterioles presented an increase in wall thickness in the MCT group. This finding is consistent with previous evidence showing that MCT-induced PAH is characterised by remodelling of pulmonary vessels [5,6,33]. It supports the current conceptualisation of PAH as a multifactorial cardiopulmonary disease involving a complex interaction between vascular dysfunction, inflammation, fibrosis, and progressive RV remodelling, rather than as an exclusively vasoconstrictive disorder. Collectively, these findings confirm the successful establishment of experimental PAH and highlight the close interplay between pulmonary vascular injury, inflammation, and RV dysfunction in this model [35,36].

Treatment with red araça extract attenuated alterations induced by MCT administration. Although red araça extract did not completely normalise pulmonary haemodynamic parameters, the MCT + red araça extract group presented lower TPVR compared with the MCT group, suggesting partial attenuation of pulmonary vascular dysfunction. Considering that increased pulmonary vascular resistance is a major determinant of RV afterload and disease progression in PAH, even partial improvement in this parameter may contribute to preservation of right heart function [1,2]. Red araça extract also prevented the increase in pulmonary arteriolar wall thickness, which may contribute to lower pulmonary vessel resistance. In addition to the haemodynamic and histological findings, treatment with red araça extract was associated with prevention of RV remodelling and dysfunction. The MCT + red araça extract group exhibited higher TAPSE, FAC, and systolic output values compared with the MCT group, indicating partial preservation of RV systolic performance. Moreover, the absence of RV hypertrophy in the MCT + red araça extract group suggests attenuation of maladaptive ventricular remodelling. These findings are particularly relevant because RV hypertrophy is one of the main predictors of poor prognosis in PAH [1,2,3,4].

Interestingly, the beneficial effects of red araça extract on RV structure and function were observed even though mPAP and RVSP remained elevated. This finding suggests that the effects of red araça extract may not be exclusively dependent on the effects in pulmonary vasculature; it could also involve direct modulation of biological pathways associated with RV inflammation, cellular stress, and adaptation. In this context, it is possible that the combined attenuation of pulmonary vessels remodelling and changes in protein expression in the RV contributed to preservation of RV performance in the MCT + red araça extract group. This integrated cardiopulmonary response suggests that the effects of red araça extract may involve simultaneous modulation of pulmonary and ventricular remodelling processes rather than isolated hemodynamic improvement alone.

The attenuation of RV remodelling and dysfunction observed in the MCT + red araça extract group was accompanied by modulated expression of proteins involved in cardiac inflammation and hypertrophy. Consistent with previous studies, the MCT group exhibited increased TLR4 protein expression in the RV, reinforcing the concept that inflammatory signalling contributes to the progression of RV remodelling in PAH [7,27]. Activation of TLR4 has been associated with increased production of pro-inflammatory mediators, fibrosis, oxidative stress, and myocardial injury, all of which may contribute to maladaptive ventricular remodelling and functional deterioration [7,10]. In contrast, there was lower TLR4 protein expression in the MCT + red araça extract group compared with the MCT group, suggesting attenuation of inflammation in the RV. In this context, attenuation of TLR4 expression may also be associated with the partial preservation of RV functional parameters observed in the MCT + red araça extract group, reinforcing the relationship between inflammation and RV dysfunction during PAH progression. Concomitantly, p-Akt/Akt ratio was markedly increased in the MCT group. This result indicates an increased Akt activation [13]. In fact, this protein signalling is recognised as an important mediator of cardiac hypertrophy under stress conditions [11,12,13]. Therefore, the increased Akt activation in the MCT group may be related with RV pathological remodelling. Regarding this, studies show that Akt activation caused a spectrum of phenotypes from moderate cardiac hypertrophy with preserved systolic function to massive cardiac dilatation and sudden death [37]. However, in the group that received the araçá extract, the activation of this enzyme is similar to the control. This result corroborates with the absence of hypertrophy in this group. In addition, administering the extract decreases the expression of XO, a pro-oxidant and pro-inflammatory enzyme. This is a positive result from araça extract administration. Regarding this, Minhas and colleagues showed that an increase in the expression of XO was demonstrated in rats with dilated cardiomyopathy and its inhibition was reported to improve cardiac remodelling [38]. Also, the decrease in the expression of this enzyme proved to be beneficial in rats subjected to acute myocardial infarction [14]. The simultaneous modulation of inflammatory and pro-hypertrophic proteins expression reinforces the possibility that the phenolic compounds present in red araça extract exert integrated multitarget biological effects during PAH progression. Considering the recognised anti-inflammatory properties of several phenolic compounds identified in red araça extract, including flavonoids and anthocyanins [39,40,41,42,43,44,45,46,47,48,49], it is possible that these bioactive molecules contributed to modulation of TLR4, XO and Akt protein expression. Previous studies have also demonstrated anti-inflammatory effects of red araça extracts in experimental models, including inhibition of inflammatory signalling pathways and reduction in cellular injury [19,20].

We also measured serum biochemical parameters to assess whether the dose of red araçá extract used in this study exerted toxicity. The only change was a slight increase in serum creatinine in the MCT and MCT + red araça extract groups. Administration of the red araçá extract did not alter markers of kidney function and liver damage, indicating a lack of toxicity.

Overall, the findings from the present study suggest that red araça extract may attenuate cardiopulmonary alterations associated with experimental PAH. In fact, natural products rich in phenolic compounds have attracted increasing attention as potential adjunctive therapeutic strategies in cardiovascular and inflammatory diseases due to their antioxidant and anti-inflammatory properties (39–49). In this context, the present findings reinforce the biological relevance of native Brazilian fruits such as red araça as potential sources of bioactive compounds with cardiopulmonary protective properties. However, this is the first study to evaluate the effect of red araça extract on PAH. More studies using other experimental models of this disease are needed to confirm these data and add translational value to these findings. Moreover, we only had modest positive effects from this intervention in some parameters and there was no change in pulmonary artery pressure and in the majority of RV hemodynamic parameters in rats with PAH. Nevertheless, the positive effects found for RV remodelling and function as well as pulmonary arterioles encourage further studies with red araça extract, which could become an adjunct in the treatment of PAH.

### Limitations

The present study has some limitations that should be acknowledged. First, although we identified alterations in TLR4, XO and Akt expression, the experimental design did not allow us to establish direct causal relationships between these molecular findings and the functional improvements observed in the MCT + red araça extract group. Second, we did not investigate dose–response effects or the specific contribution of individual bioactive compounds present in red araça extract. Third, we only tested one dose of the extract. Fourth, we did not quantify each component of the extract: we only identified the compounds present as well as the total phenolic content; we used the latter to determine the dose administered to the rats. Fifth, we did not include a group treated with red araçá extract but not MCT. This group could provide information about the effects of this extract on the hearts of healthy animals. Finally, we used only male rats because in females, it is necessary to assess the stage of the oestrous cycle to ensure that hormonal changes do not affect the results. We used males to make the experiment more manageable, given that this is the first time red araçá extract has been tested. Because PAH is more common in women than in men, it is important to determine whether female rats with PAH show the same responses to the administration of red araçá extract as male rats.

## 5. Conclusions

In conclusion, red araça extract attenuated important cardiopulmonary alterations associated with MCT-induced PAH. Treatment with red araça extract was associated with partial preservation of RV function, attenuation of RV hypertrophy, reduction in pulmonary vascular resistance, modulation of TLR4, XO and Akt protein expression, and prevention of pulmonary arteriole remodelling. Collectively, these findings suggest that red araça extract may exert cardiopulmonary protective effects in an experimental model of PAH, possibly involving modulation of inflammatory and pro-hypertrophic proteins expression. To the best of our knowledge, this is the first study to investigate the effects of red araça extract in experimental PAH, highlighting the potential relevance of this native Brazilian fruit as a source of bioactive compounds with possible cardiopulmonary benefits. However, additional studies are necessary to further elucidate the mechanisms involved and to better determine the translational potential of red araça extract in PAH.

## Figures and Tables

**Table 1:** The nutritional profile of the rat chow used in this study.

	Nutritional Information
Product	NUVILAB CR-1 AUTOCLAVABLE (Code: 100110075); laboratory rodent feed for mice and rats; net weight: 10.00 kg
Main Ingredients	Soybean meal, wheat meal, corn, refined soybean oil, limestone (calcitic/dolomitic), sodium chloride, monocalcium phosphate, DL-methionine, L-lysine HCl, biotin, choline chloride, niacin, calcium pantothenate, vitamins A, B1, B2, B6, B12, D3, E, K3, folic acid, calcium iodate, sodium selenite, cobalt, copper, iron, manganese and zinc sulfates, calcium propionate, potassium sorbate, BHT.
Nutritional profile	Moisture ≤ 120 g/kg; crude protein ≥ 220 g/kg; ether extract ≥ 40 g/kg; Ash ≤ 90 g/kg; crude fibre ≤ 70 g/kg; calcium 10–14 g/kg; phosphorus ≥ 8 g/kg
Vitamins	A 25,500 IU/kg; D3 2100 IU/kg; E 60 IU/kg; K3 12.5 mg/kg; B1 14.4 mg/kg; B2 11 mg/kg; B6 12 mg/kg; B12 60 µg/kg; niacin 60 mg/kg; calcium pantothenate 112 mg/kg; folic acid 6 mg/kg; biotin 0.26 mg/kg; choline 2400 mg/kg
Minerals and amino Acids	Sodium 2700 mg/kg; iron 50 mg/kg; manganese 60 mg/kg; zinc 60 mg/kg; copper 10 mg/kg; iodine 2 mg/kg; selenium 0.05 mg/kg; cobalt 1.5 mg/kg; fluorine ≤ 80 mg/kg; lysine 14 g/kg; methionine 5000 mg/kg

**Table 2:** Major bioactive compounds identified in red araça extract.

Compound	tR (min) ^a^	Molecular Formula	Adduct [M–H]	Error (ppm)	MS^2^ Fragments	Level ^b^
Citric acid	0.4	C_6_H_8_O_7_	191.0206	−4.6	87.0082; 191.0213	1
Galloyl glucoside	1.1	C_13_H_16_O_10_	331.068	−2.8	169.0141, 271.0476, 125.0232, 211.0226, 151.0007	2
Galloyl glucoside II	1.2	C_13_H_16_O_10_	331.0675	−1.4	169.0149; 271.0467, 125.0243,	2
Galloyl glucoside V	1.5	C_13_H_16_O_10_	331.0673	−0.8	169.0152, 125.0256, 271.0461	2
Citric acid	1.9	C_6_H_8_O_7_	191.0204	−5.3	85.0293; 87.0079; 191.0208	2
Vanillic acid hexoside derivative	2.0	n.d.	536.1131	n.d.	167.0354, 152.0117, 123.0454	2
HHDP-hexoside	2.5	n.d.	481.0992	n.d.	319.0462, 275.0464,	2
Taxifolin hexoside	2.6	C_21_H_22_O_12_	465.1038	0.0	303.0505, 194. 9935	2
Gallocatechin derivative	2.7	n.d.	425.1454	n.d.	305.10325	2
Gallocatechin	2.8	C_15_H_14_O_7_	305.0707	−13.2	305.07	2
Ellagic derivative	2.9	n.d.	381.1232	n.d.	300.9941	2
Isofraxidin hexoside derivative	3.0	Nd n.d.	559.1671	n.d.	383.1353, 221.0824, 193.0825,	2
Quercetin glucuronide	3.0	C_21_H_18_O_13_	477.0675	−0.3	301.0350, 283.0262, 1,510,041	2
Eriodicytol glucoside	3.2	C_21_H_22_O_11_	449.1093	−0.9	287.0554	2
Quercetin-malonyl-glucoronide	3.4	n.d.	725.1928	n.d.	549.1611, 313.0566, 235.0978, 175.0252	2
Abscisic acid	3.6	C_15_H_20_O_4_	263.1293	−1.6	219.1391, 203.1023, 189.0901	2
Kaempferol	4.1	C_15_H_10_O_6_	285.0405	0.0	285.0407	1
Daidzein	4.3	C_15_H_10_O_4_	253.0511	−1.9	223.0385; 224.0470; 253.0491; 208,0473	2
Pinocembrine	4.8	C_15_H_12_O_4_	255.0656	2.8	255.0670; 171.0451; 213.0536; 151.0046;	2
Tryptophan	2.1	C_11_H_12_N_2_O_2_	205.0973 ^b^	−0.6	118,0648; 142, 0648; 144,0797; 146,0599	2
Cyanidin-3-*O*-galactoside	2.3	C_21_H_21_O_11_	449.1084 ^b^	−1.1	287.0554	2

^a^ Retention time on the C18 Acquity (1.8 μm) UPLC column and solvent: gradient of 0.1% formic acid in water and acetonitrile with 0.1% formic acid. n.d. = not detected. ^b^ The molecule is ionised [M]+, Identification confidence level according to Schymanski et al. [22].

**Table 3:** Right ventricular hemodynamic parameters.

Parameter	Control	MCT	MCT + Red Araça Extract
RVEDP (mmHg)	2.72 ± 1.28	2.00 ± 1.46	2.48 ± 1.75
RVSP (mmHg)	28.86 ± 4.47	39.38 ± 7.91 **^a^**	42.50 ± 7.38 **^a^**
+dP/dt (mmHg/s)	1234.36 ± 396	1686.78 ± 269.27	1451.88 ± 452.28
−dP/dt (mmHg/s)	792.77 ± 198	1257.33 ± 223 **^a^**	1107.25 ± 323 **^a^**
TPVR (dyn·s·cm^−5^)	2.50 ± 0.40	4.26 ± 0.83 **^a^**	3.40 ± 0.67 **^a^^,^^b^**
mPAP (mmHg)	19.60 ± 2.72	26.02 ± 4.8 **^a^**	27.93 ± 4.5 **^a^**

Hemodynamic analyses were performed in the control, monocrotaline (MCT), and MCT + red araça extract groups. Representative hemodynamic parameters of right ventricular systolic pressure (RVSP) (*p* = 0.0056), right ventricular end-diastolic pressure (RVEDP), the right ventricular relaxation index (−dP/dt) (*p* = 0.0155), the right ventricular contractility index (+dP/dt), total pulmonary vascular resistance (TPVR) (*p* = 0.0010), and mean pulmonary artery pressure (mPAP) (*p* = 0.0056) are shown. The data are presented as mean ± standard deviation (*n* = 10 rats per group). ^a^, *p* < 0.05 versus the control group; ^b^, *p* < 0.05 versus the MCT group.

**Table 4:** Serum parameters of kidney function and liver injury.

Parameter	Control	MCT	MCT + Red Araça Extract
Creatinine (mg/dL)	0.42 ± 0.02	0.48 ± 0.04 **^a^**	0.46 ± 0.03 **^a^**
Urea (mg/dL)	49.54 ± 5.3	52.36 ± 6.6	51.40 ± 7.1
ALT (U/L)	17.45 ± 5.8	17.90 ± 4.3	14.90 ± 4.06
AST (U/L)	152 ± 543	199 ± 72	147 ± 20

ALT, alanine aminotransferase; AST, aspartate aminotransferase; MCT, monocrotaline. The data are presented as the mean ± standard deviation (*n* = 10 rats per group). ^a^, *p* < 0.05 versus the control group.

## Data Availability

The data presented in this study are available on request from the corresponding author due to privacy reasons.

## Abbreviations

−dP/dt	right ventricle relaxation index
+dP/dt	right ventricle contractility index
Akt	protein kinase B
ANOVA	analysis of variance
ERK	extracellular signal-regulated kinase
FAC	fractional area change
HE	haematoxylin and eosin
HPLC	high-performance liquid chromatography
LV	left ventricle
MCT	monocrotaline
MMP	metalloproteinases
mPAP	mean pulmonary artery pressure
PAH	pulmonary artery hypertension
QTOF-MS	quadrupole-time-of flight mass spectrometry
ROS	reactive oxygen species
RV	right ventricle
RVEDP	right ventricular end-diastolic pressure
RVSP	right ventricular systolic pressure
TAPSE	tricuspid annular plane systolic excursion
TBARS	thiobarbituric acid reactive substances
TPVR	total pulmonary vascular resistance
TLR4	Toll-like receptor 4
UHPLC	ultra-high-performance liquid chromatography
XO	xanthine oxidase
